# A supramolecular system mimicking the infection process of an enveloped virus through membrane fusion

**DOI:** 10.1038/s41598-023-47347-7

**Published:** 2023-11-15

**Authors:** Hiroto Furukawa, Yuuna Kimura, Hiroshi Inaba, Kazunori Matsuura

**Affiliations:** 1 Department of Chemistry and Biotechnology, Graduate School of Engineering, Tottori University, 4-101 Koyama-Minami, Tottori, 680-8552 Japan; 2 Center for Research on Green Sustainable Chemistry, Tottori University, 4-101 Koyama-Minami, Tottori, 680-8552 Japan

**Keywords:** Biochemistry, Supramolecular chemistry

## Abstract

Membrane fusion is an essential step for the entry of enveloped viruses, such as human immunodeficiency virus and influenza virus, into the host cell, often triggered by the binding of membrane proteins on the viral envelope to host cell membrane. Recently, external stimuli was shown to trigger membrane fusion in an artificial system. Direct observation of artificial membrane fusion using a giant unilamellar vesicle (GUV), which is similar in size to a cell, is useful as a biological model system. However, there are no model systems for studying membrane fusion of enveloped viruses with host cells. Here, we report a supramolecular model system for viral entry into a GUV or cell through membrane fusion. The system was constructed by complexing a cationic lipid bilayer on an anionic artificial viral capsid, self-assembled from viral β-annulus peptides. We demonstrate that the cationic enveloped artificial viral capsid electrostatically interacts with the anionic GUV or cell, and the capsid enters the GUV or cell through membrane fusion. The model system established in this study will be important for analyzing membrane fusion during infection of a natural virus.

## Introduction

Membrane fusion is fundamental to biological processes, such as intracellular compartmentalization, cell growth, hormone secretion, neurotransmission, and oocyte fertilization^1,2^. Membrane fusion proceeds with the gradual merging of two opposite membranes into a continuous lipid bilayer, causing the mixing of lipids and contents^3^. Membrane fusion is an essential step during infection of host cells by enveloped viruses, and is often triggered by the binding of viral membrane proteins to host cell membrane^4^. For example, glycoprotein gp41 on the envelope of human immunodeficiency virus (HIV) promotes membrane fusion for entry into the host cell^5,6^. Influenza virus is taken up through endocytosis, which is triggered by the binding of hemagglutinin on the envelope membrane to sialyl oligosaccharides on host cell surface; membrane fusion proceeds by partial cleavage in the trans-Golgi network, and the capsid enters the cell^7^. Thus, membrane fusion induced by molecular recognition between membrane proteins on the envelope and the host cell membrane is important for enveloped virus infection.

Membrane fusion between cells can be triggered by external stimuli, such as laser irradiation^8^ and nanoheating^9^. Artificial membrane fusion systems have been developed between liposomes triggered by reconstituted protein^10,11^, fusogenic peptide^12–14^, macromolecule^15,16^, and fusogenic DNA^17^. Direct observation of artificial membrane fusion using a giant unilamellar vesicle (GUV), which is similar in size to a cell, is useful as a model system to examine the physicochemical mechanism of membrane fusion. Riske et al. analyzed electrostatic interactions between a cationic large unilamellar vesicle (LUV, ~ 140 nm) containing 1,2-dioleoyl-3-trimethylammonium-propane (DOTAP) and an anionic GUV (> 20 μm) containing 1-palmitoyl-2-oleoyl-*sn*-glycero-3-phospho-(1′-rac-glycerol) (sodium salt) (POPG), and found that the more anionic lipid POPG (in GUV) induced faster and more efficient membrane fusion^18^. Other artificial membrane fusion processes using GUVs have been reported to be induced by an electrical pulse^19,20^, external stimulus-responsive lipids^21,22^, fusogenic peptides^23,24^, fusogenic DNA^25^, and optically heated gold nanoparticles^26^. However, to our knowledge, there are no model systems for studying membrane fusion of enveloped virus with host cell. Membrane fusion of an enveloped viral model into a GUV or cell is a promising artificial system to understand the infection mechanism of enveloped viruses.

As candidates for an artificial viral model, virus-like particles (viral capsid)^27–34^ and protein nanocapsules^35–39^, self-assembled from artificially designed proteins, have attracted great attention due to their potential as carriers in drug delivery systems^40–44^, nanoreactors^45–48^, and intracellular trafficking material^49–52^. However, to our knowledge, there is no example of an artificial viral model to analyze viral entry into a GUV or cell by membrane fusion. We have demonstrated that a 24-residue β-annulus peptide (INHVGGTGGAIMAPVAVTRQLVGS) involved in the formation of the dodecahedral internal skeleton of tomato bushy stunt virus spontaneously self-assembles into a hollow artificial viral capsid (size: 30–50 nm) in water^53,54^. The artificial viral capsid encapsulated various guest molecules and its external surface could be modified with functional molecules^54–59^. Recently, we constructed an enveloped artificial viral capsid by complexing an anionic artificial viral capsid, self-assembled from β-annulus-EE peptide (INHVGGTGGAIMAPVAVTRQLVGSEE), with a cationic lipid bilayer, comprising DOTAP and 1,2-dioleoyl-*sn*-glycero-3-phosphocholine (DOPC)^60^, which can be equipped with a membrane protein^61^ and antigen and adjuvant^62^ on the envelope. In this study, we aim to construct a supramolecular model system for internal entry into a GUV or cell through membrane fusion using an enveloped artificial viral capsid. The interaction of the enveloped artificial viral capsid, containing a cationic lipid, with a GUV or cell, and the internal entry of the capsid by membrane fusion were analyzed by confocal laser scanning fluorescence microscopy (CLSM).

## Results and discussion

### Construction of multifluorescence-labeled enveloped artificial viral capsids

**Figure 1:**
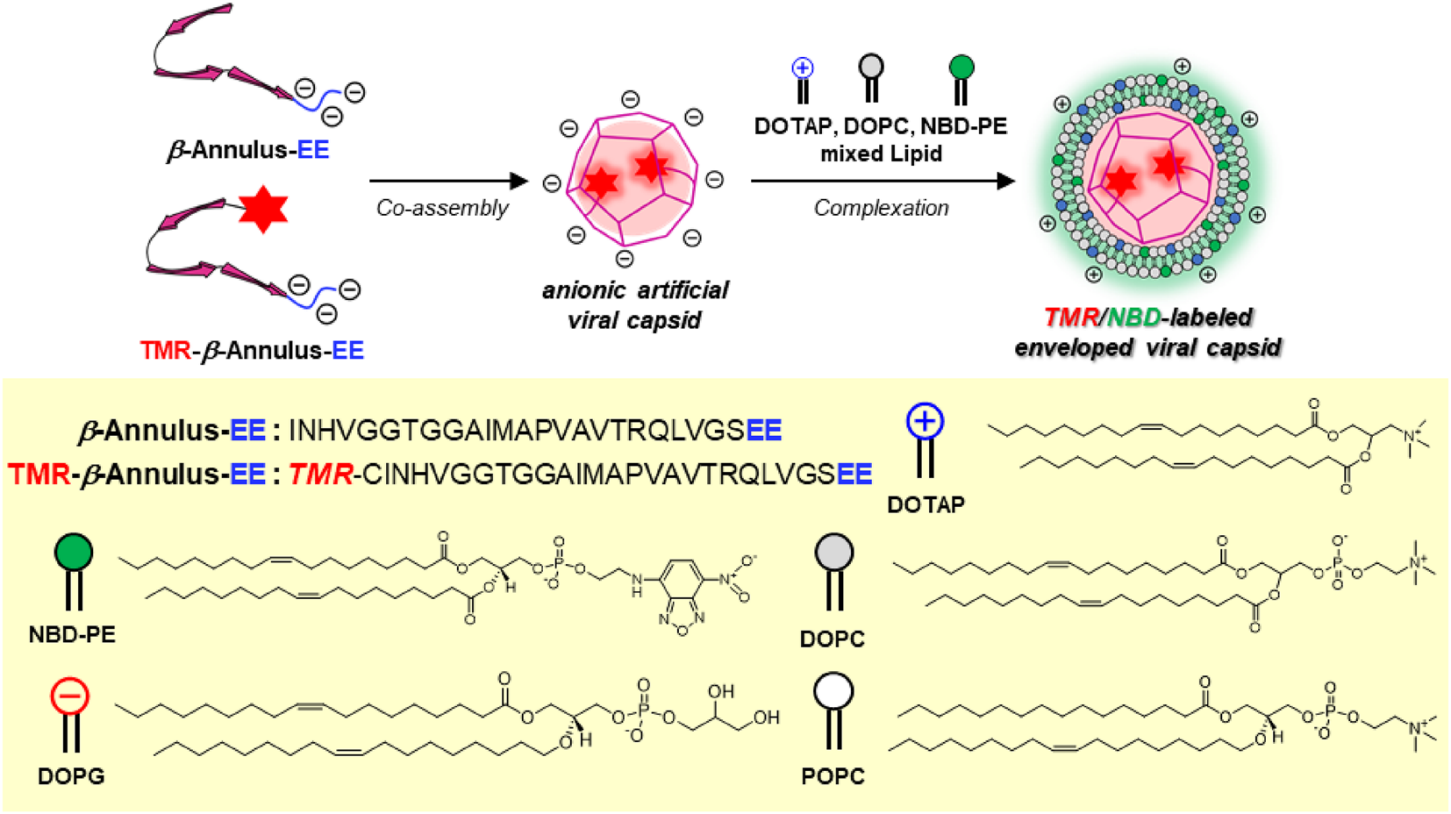
Schematic illustration showing construction of TMR/NBD-labeled enveloped viral capsid.

As a scaffold for multifluorescence-labeled enveloped artificial viral capsid, we constructed artificial viral capsids possessing an anionic exterior and an interior modified with fluorescent dyes (Fig. 1). Because the C-terminal side of β-annulus peptide is directed toward the outside of the artificial viral capsid^63^, we designed a 26-residue β-annulus-EE peptide with two anionic amino acids (Glu) at the C-terminal side (INHVGGTGGAIMAPVAVTRQLVGSEE)^60^. β-Annulus-EE peptide was synthesized using the Fmoc solid-phase method^64^, purified by reverse-phase HPLC, and confirmed by MALDI-TOF MS (m/z = 2564 [M + H]^+^) (Fig. S1). β-annulus-EE peptide, modified with fluorescent dye tetramethylrhodamine (TMR) at the N-terminal side (TMR-β-annulus-EE peptide) directed toward the inside of the capsid, was synthesized by Michael addition of N-terminal Cys residue from Cys-β-annulus-EE peptide (CINHVGGTGGAIMAPVAVTRQLVGSEE) to TMR-maleimide (m/z = 3149 [M + 2]^+^) (Fig. S2).

**Figure 2:**
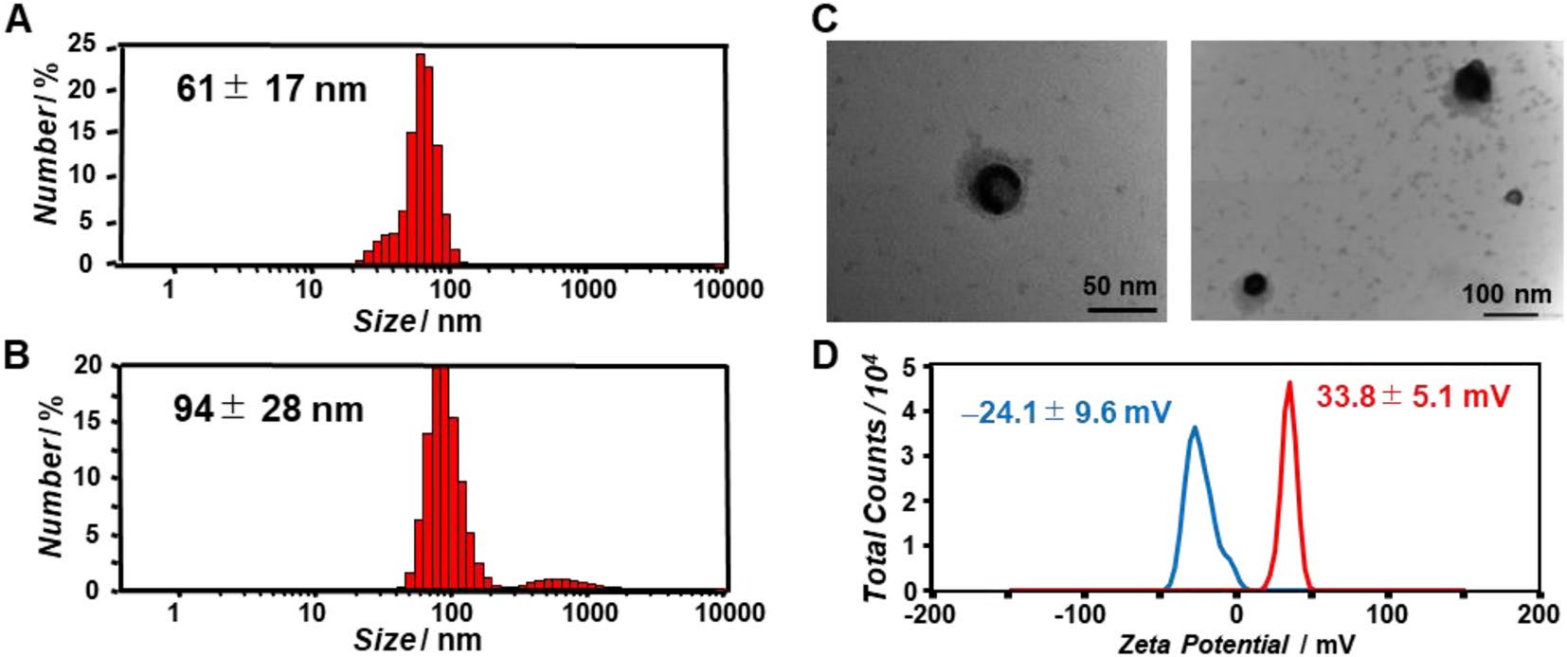
Size distributions of (**A**) TMR-labeled capsid (90 μM β-annulus-EE peptide, 10 μM TMR-β-annulus-EE peptide) and (**B**) TMR/NBD-labeled enveloped viral capsid (90 μM β-annulus-EE peptide, 10 μM TMR-β-annulus-EE peptide, 300 μM DOTAP, 2985 μM DOPC, and 15 μM NBD-PE) were obtained using dynamic light scattering in 10 mM phosphate buffer (pH 7.0) containing 1% dimethyl sulfoxide (DMSO) at 25 °C. (**C**) TEM images of TMR/NBD-labeled enveloped viral capsid stained with EM stainer. (**D**) ζ-potential of TMR-labeled capsid (blue) and TMR/NBD-labeled enveloped viral capsid (red) at pH 7.0.

TMR-β-annulus-EE and β-annulus-EE peptides were co-assembled at a molar ratio of 1:9 in 10 mM phosphate buffer (pH 7.0) to prepare TMR-labeled capsid (90 μM β-annulus-EE peptide, 10 μM TMR-β-annulus-EE peptide). Dynamic light scattering (DLS) analysis showed that the diameter of TMR-labeled capsid was 61 ± 17 nm (Fig. 2A). The anionic TMR-labeled capsid was complexed with lipid bilayers containing the cationic lipid DOTAP, zwitterionic lipid DOPC, and NBD-labeled lipid 1,2-dioleoyl-*sn*-glycero-3-phosphoethanolamine-*N*-(7-nitro-2-1,3-benzoxadiazol-4-yl) (NBD-PE) at a charge ratio of 1:1 (molar ratio DOTAP:DOPC:NBD-PE = 1.0:9.95:0.05) by the hydration method (Fig. 1). Size distribution of the complex in 10 mM phosphate buffer was 94 ± 28 nm (Fig. 2B). The increase in particle size upon complexation reflects the thickness of the lipid coating on the surface of the anionic capsid. Transmission electron microscopy (TEM) analysis showed spherical structures (diameter = 70–100 nm), coated with a fringe structure that appeared to be a lipid bilayer (Fig. 2C). The ζ-potential of the anionic artificial viral capsid was − 24.1 ± 9.6 mV (Fig. 2D, blue), whereas that of the enveloped artificial viral capsid complexed with DOTAP/DOPC/NBD-PE mixed lipid was 33.8 ± 5.1 mV (Fig. 2D, red), indicating that the cationic lipid bilayer coated the surface of the anionic artificial capsid. Thus, we constructed TMR/NBD-labeled enveloped artificial viral capsid by complexing the anionic capsid with a cationic lipid bilayer.

### Interaction between enveloped artificial viral capsids and GUVs

**Figure 3:**
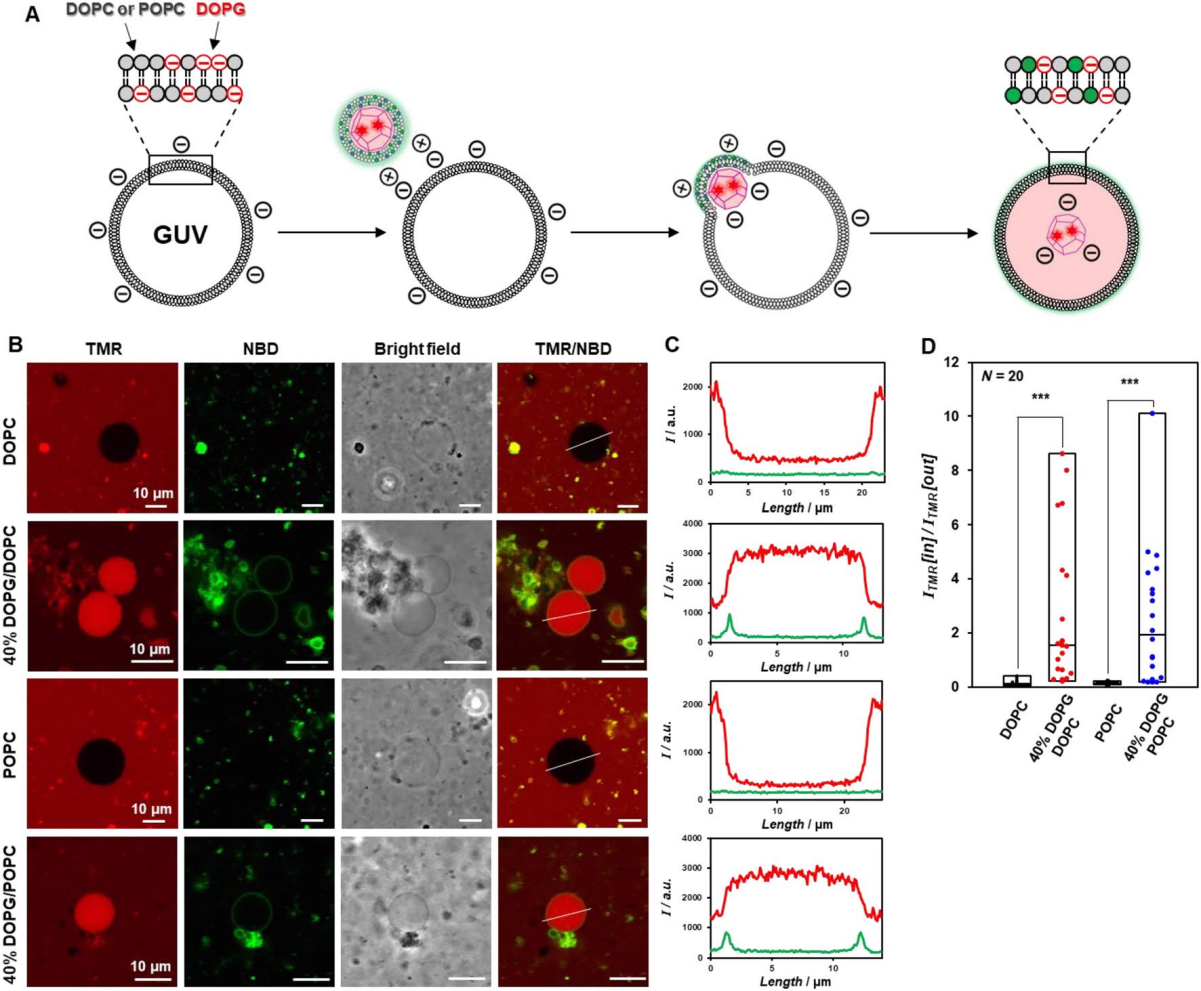
(**A**) Schematic illustration showing interaction between TMR/NBD-labeled enveloped viral capsid and anionic GUVs. (**B**) CLSM images of DOPC GUV (1 mM DOPC), 40% DOPG/DOPC GUV (0.4 mM DOPG, 0.6 mM DOPC), POPC GUV (1 mM POPC), and 40% DOPG/POPC GUV (0.4 mM DOPG, 0.6 mM POPC) interacting with TMR/NBD-labeled enveloped viral capsid (45 μM β-annulus-EE peptide, 5 μM TMR-β-annulus-EE peptide, 150 μM DOTAP, 1492.5 μM DOPC, and 7.5 μM NBD-PE). (**C**) The fluorescence intensity profiles of (**B**) (red; TMR, green; NBD). (**D**) Box plot of the fluorescence intensity ratio of TMR inside/outside of each GUV (*N* = 20). *P*-value was calculated by one-way analysis of variance followed by Dunnett’s post hoc test (***P < 0.001).

We evaluated how multifluorescence-labeled enveloped artificial viral capsids interact with GUVs by fluorescence imaging. GUVs comprising only DOPC or 1-palmitoyl-2-oleoylphosphatidylcholine (POPC) and mixed GUVs containing 40 mol% anionic lipid 1,2-dioleoyl-*sn*-glycero-3-phospho-rac-(1-glycerol) (DOPG) of total lipids (40% DOPG/DOPC or 40% DOPG/POPC) were prepared by the hydration method. Since the ratio of anionic lipids in typical mammalian cell membranes is approximately 30–50%^65^, we prepared GUVs containing 40% DOPG/DOPC or DOPG/POPC, which are commonly used for direct observation of membrane fusion^18,21–26^. GUV suspensions in 10 mM phosphate buffer (pH 7.0) were incubated with a solution of TMR/NBD-labeled enveloped capsid in the same buffer at a volume ratio of 1:1 for 1 h and observed by CLSM (Fig. 3A). TMR-derived fluorescence was strong inside the GUV containing anionic DOPG and weak inside the GUV comprising DOPC or POPC (Fig. 3B). Interestingly, the GUV containing anionic DOPG showed NBD-derived fluorescence on the GUV membrane and increased TMR-derived fluorescence inside the GUV (Fig. 3C). These results indicate that because the cationic enveloped capsid fused with the anionic GUV, the envelope lipids on the GUV membrane internalized the capsid only. By contrast, no increase in TMR-derived fluorescence was observed inside DOPC or POPC GUVs. It is probable that the membrane fusion induced the electrostatic relaxation between the anionic capsid and the cationic envelope within the enveloped capsid. As the result, a part of the anionic capsid should be exposed inside the GUV. We envisage that electrostatic repulsion between the exposed anionic surface of capsid and the anionic lipids on the GUV membrane might promote internalization of the capsid inside the GUV. And the lipids derived from the envelope should remain in the GUV membrane due to the significant hydrophobic interaction in the membrane.

GUVs without green or red internalized fluorescence, and that with green fluorescence in the membrane and no internal fluorescence might be caused by diversity in the interaction between the enveloped capsid and each GUV due to differences in each GUV particle size and membrane composition (Fig. 3B, Figs. S3–S6). For GUVs without green fluorescence in the membrane but with red fluorescence inside, the NBD fluorescence might be quenched due to fluorescence resonance energy transfer (FRET) between the TMR labeled on capsid inside the GUV and the NBD on the membrane. The fluorescence intensity ratio of TMR inside/outside (I_TMR_ [in]/I_TMR_ [out]) of each GUV significantly increased in the presence of anionic DOPG (Fig. 3D, Figs. S3–S6). For GUVs with anionic DOPG, fluorescence intensity ratio of TMR inside/outside increased with decreasing GUV surface area, whereas for GUVs without anionic DOPG, the ratio did not increase significantly with decreasing GUV surface area. The correlation between GUV surface area and inside/outside fluorescence intensity ratio were roughly approximated by a power law equation (Fig. S7). These results suggest that anionic GUV membranes possessing higher curvature promote membrane fusion with the cationic enveloped capsid.

**Figure 4:**
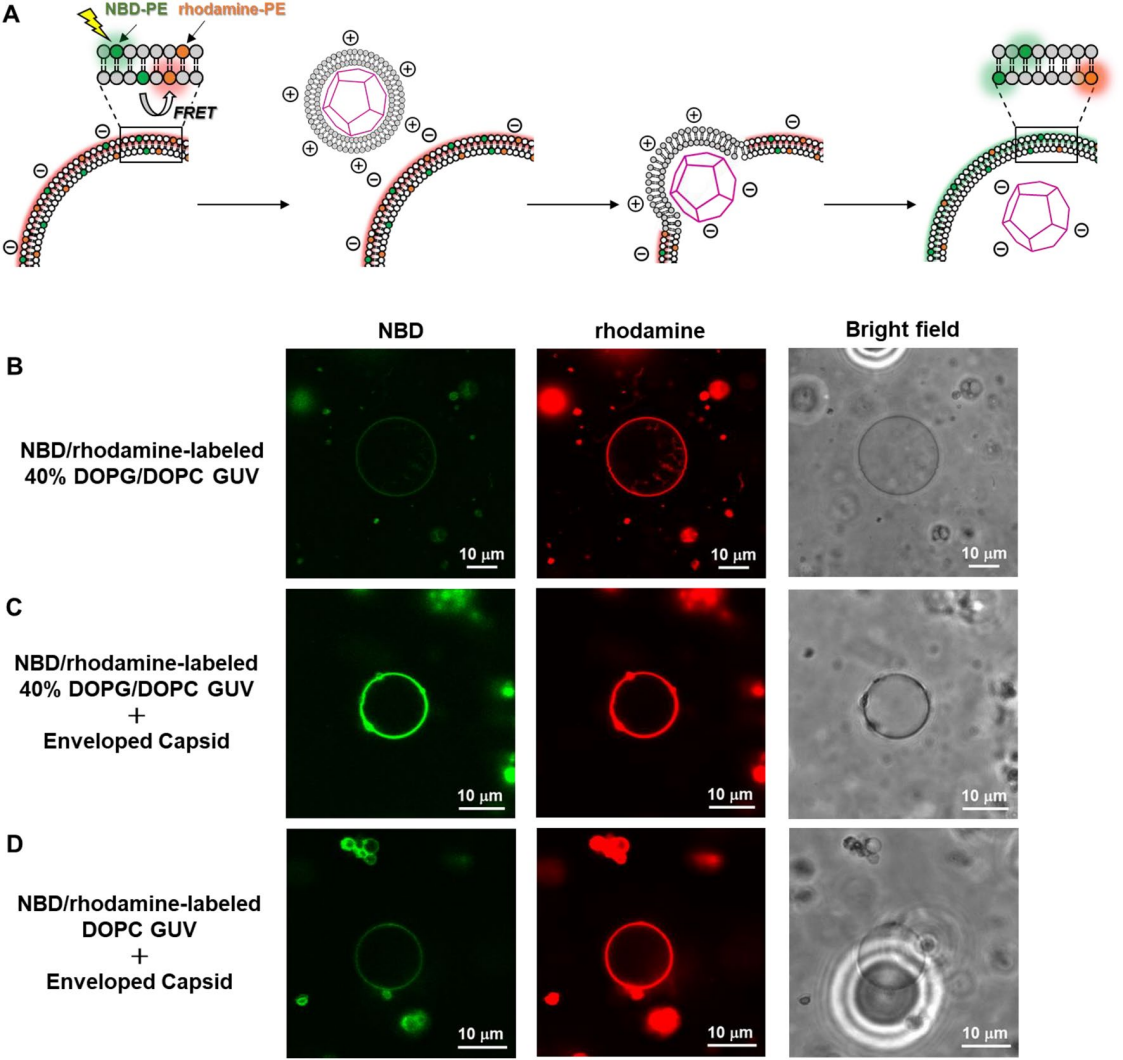
(**A**) Schematic illustration of lipid-mixing assay between NBD/rhodamine-labeled 40% DOPG/DOPC GUV and enveloped capsid. (**B**) CLSM image of NBD/rhodamine-labeled 40% DOPG/DOPC GUV (0.6 mM DOPC, 0.4 mM DOPG, 7.5 μM NBD-PE, 15 μM rhodamine-PE). (**C**) CLSM image of the NBD/rhodamine-labeled 40% DOPG/DOPC GUV interacting with enveloped capsid (50 μM β-annulus-EE peptide, 150 μM DOTAP, and 1500 μM DOPC). (**D**) CLSM image of NBD/rhodamine-labeled DOPC GUV (1 mM DOPC, 7.5 μM NBD-PE, 15 μM rhodamine-PE) interacting with the enveloped capsid (50 μM β-annulus-EE peptide, 150 μM DOTAP, and 1500 μM DOPC).

To directly confirm the entry of envelope components into the GUV by membrane fusion, we observed CLSM images of fluorescence-labeled GUVs before and after the membrane fusion based on lipid-mixing assay^66^ (Fig. 4A). We prepared NBD/rhodamine-labeled 40% DOPG/DOPC GUVs, in which the NBD-derived fluorescence quenched by FRET from NBD to rhodamine (Fig. 4B). When the enveloped capsid was added to the NBD/rhodamine-labeled 40% DOPG/DOPC GUV, the quenched NBD-derived fluorescence was recovered by decrease in FRET efficiency through the entry of envelope-derived lipids into the GUV membrane (Fig. 4C). In contrast, when the enveloped capsid was added to NBD/rhodamine-labeled 40% DOPC GUV, fluorescence recovery was hardly observed (Fig. 4D). These results strongly support entry of the cationic enveloped capsids into the anionic GUVs by membrane fusion.

**Figure 5:**
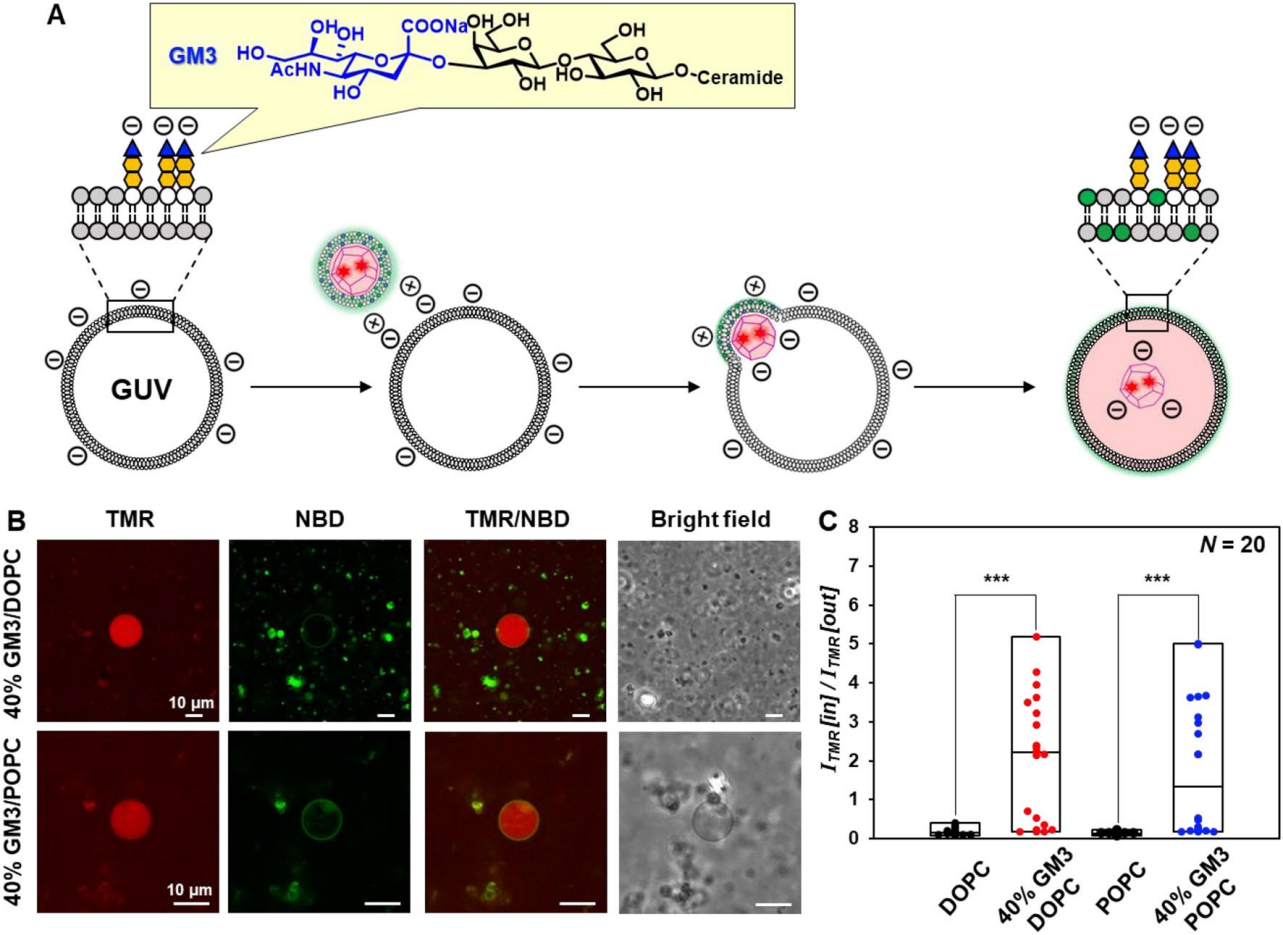
(**A**) Schematic illustration of TMR/NBD-labeled enveloped viral capsid with a GUV containing glycolipid. (**B**) CLSM images of 40% GM3/DOPC GUV (0.4 mM GM3, 0.6 mM DOPC) and 40% GM3/POPC GUV (0.4 mM GM3, 0.6 mM POPC) interacting with TMR/NBD-labeled enveloped viral capsid (45 μM β-annulus-EE peptide, 5 μM TMR-β-annulus-EE peptide, 150 μM DOTAP, 1492.5 μM DOPC, and 7.5 μM NBD-PE). (**C**) Box plot showing fluorescence intensity ratio of TMR inside/outside of each GUV (*N* = 20). Box plots of DOPC or POPC GUV using the values from Fig. 3D. *P*-value was calculated by one-way analysis of variance followed by Dunnett’s post hoc test (***P < 0.001).

In addition, we evaluated the interaction between the cationic enveloped capsid and a GUV containing the anionic lipid ganglioside monosialate 3 (GM3), which is present on cell membrane surface. When the TMR/NBD-labeled enveloped capsid was added to a GUV containing 40% GM3, TMR-derived fluorescence inside the GUV was qualitatively comparable to that of the DOPG-containing GUV (Fig. 5A,B). Inside/outside fluorescence intensity ratio was significantly higher for GUVs with GM3 than GUVs without GM3 (Fig. 5C, Figs. S8, S9). These results suggest that enveloped capsids interact with anionic glycolipids on the plasma membrane and get internalized.

### Interaction between enveloped artificial viral capsids and cells

**Figure 6:**
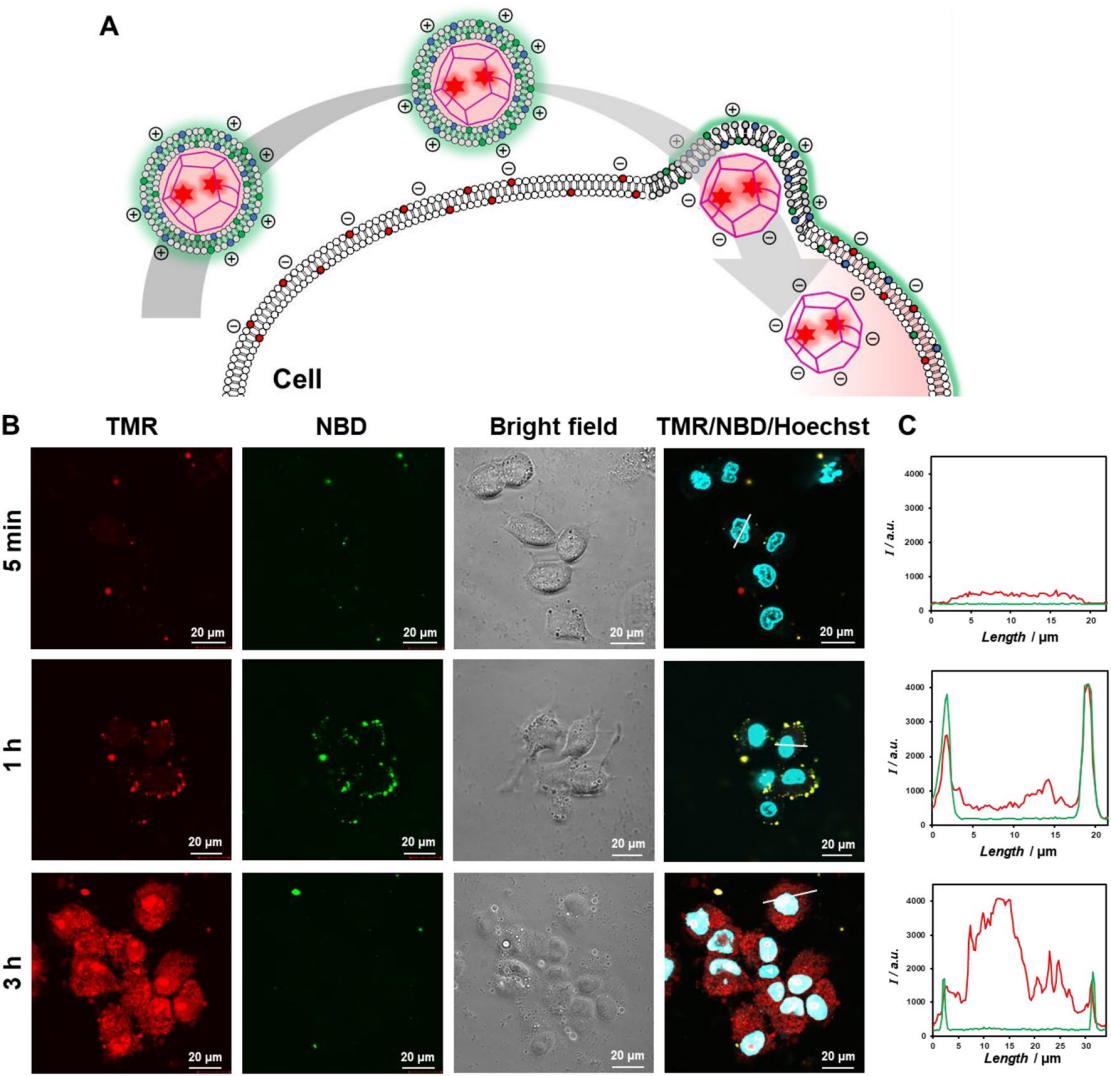
(**A**) Schematic illustration of enveloped viral capsid entry into HepG2 cells. (**B**) CLSM images of HepG2 cells incubated with TMR/NBD-labeled enveloped viral capsid (45 μM β-annulus-EE peptide, 5 μM TMR-β-annulus-EE peptide, 150 μM DOTAP, 1492.5 μM DOPC and 7.5 μM NBD-PE) for 5 min, 1 h, and 3 h. Nuclei were stained with Hoechst 33342. (**C**) The fluorescence intensity profiles of (**B**) (red; TMR, green; NBD).

We evaluated whether enveloped capsids are taken up by cells through membrane fusion (Fig. 6A). Human hepatocellular carcinoma-derived HepG2 cells have an anionic plasma membrane surface containing the anionic lipids DOPS, DOPG, and DOPI, which comprise approximately 40% of the total phospholipids^67^. TMR/NBD-labeled enveloped capsids were incubated with HepG2 cells for 5 min, 1 h, and 3 h, and intracellular fluorescence was observed by CLSM (Fig. 6B,C). After 5 min, no fluorescence was observed inside the cell or on the cell surface, whereas after 1 h, yellow fluorescent dots colocalizing TMR and NBD were observed on the cell surface. Remarkably, after 3 h, strong TMR fluorescence was observed throughout the cell. The fluorescence intensity profile of TMR and NBD after 5 min showed no significant increase in fluorescence intensity on the cell surface or inside the cell. After 1 h, TMR- and NBD-derived fluorescence intensity increased on the cell surface. After 3 h, NBD-derived fluorescence was visible on the cell surface, and TMR-derived fluorescence intensity dramatically increased inside the cell. By contrast, when anionic capsids or enveloped capsids coated with DOPC (without DOTAP) were added to HepG2 cells, only weak TMR fluorescence was observed inside the cell (Fig. S10). Thus, the enveloped capsids entered the cells through membrane fusion mediated by electrostatic interactions.

**Figure 7:**
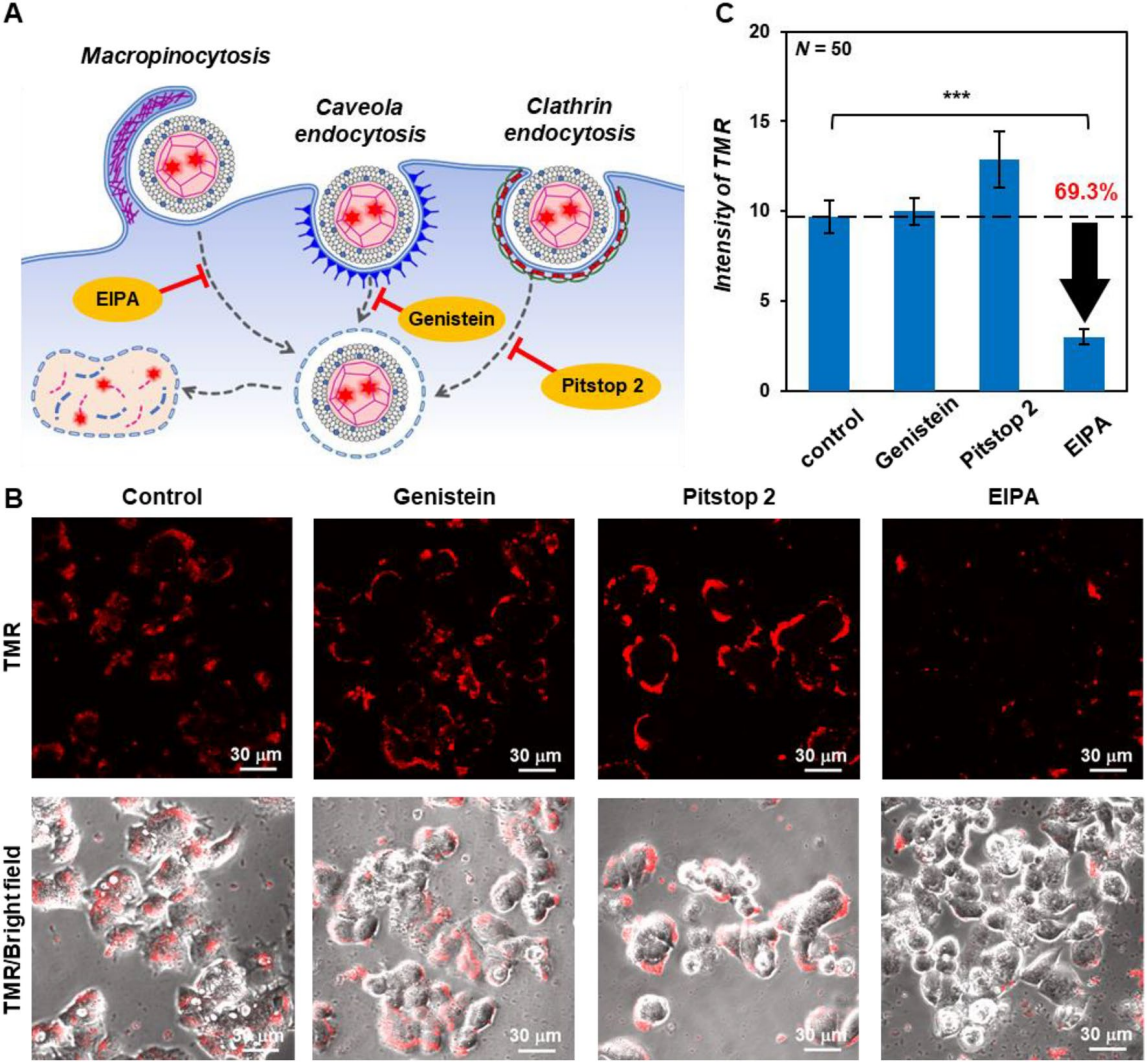
(**A**) Schematic illustration showing uptake inhibition by endocytosis inhibitors, (**B**) CLSM images, and (**C**) intensity of TMR in HepG2 cells untreated and treated with endocytosis inhibitors (80 μM EIPA, 160 μM genistein, 20 μM Pitstop 2) after addition of the TMR-labeled enveloped viral capsid (45 μM β-annulus-EE peptide, 5 μM TMR-β-annulus-EE peptide, 150 μM DOTAP, and 1500 μM DOPC). *P*-value was calculated by one-way analysis of variance followed by Dunnett’s post hoc test (***P < 0.001).

To rule out cellular uptake of the enveloped capsids by endocytosis, we analyzed the effect of endocytosis inhibitors on cellular uptake (Fig. 7A). In this experiment, TMR-labeled enveloped capsids with diameter of 121 ± 58 nm (Fig. S11) were used instead of the TMR/NBD-labeled enveloped capsids since it does not have to track membrane components. HepG2 cells were pretreated with ethylisopropyl amiloride 3-amino-*N*-(aminoiminomethyl)-6-chloro-5-[ethyl(1-methylethyl)amino]-2-pyrazinecarboxamide) (EIPA), inhibitor of micropinocytosis; genistein, inhibitor of caveolae-mediated endocytosis; and Pitstop 2, inhibitor of clathrin-mediated endocytosis^68^, incubated with TMR-labeled enveloped capsids for 3 h, and analyzed using CLSM. Strong TMR fluorescence was observed on the surface and inside of genistein- and Pitstop 2-treated cells, as well as untreated cells, whereas fluorescence intensity was significantly decreased in EIPA-treated cells (Fig. 7B). After addition of the inhibitors, TMR-derived fluorescence was observed mainly on the cell membrane surface. Probably, the inhibitors might also inhibited membrane fusion. Compared to untreated cells, genistein- and Pitstop 2-treated cells showed minimum decrease and EIPA-treated cells showed significant decrease (to 69.3%) in fluorescence intensity on the cell surface and inside the cell (Fig. 7C). These results indicate that enveloped capsids are taken up into cells through not only membrane fusion, but also micropinocytosis. Because these pathways are dependent on interactions with the cell membrane, the interaction between a cationic lipid-containing enveloped capsid and an anionic cell membrane may trigger uptake.

## Conclusion

In conclusion, we have developed a supramolecular model system that mimics the entry of natural enveloped viruses into the host cell. Electrostatic interactions between cationic enveloped artificial viral capsids and GUVs containing anionic lipids or glycolipids induced membrane fusion to internalize the capsid only. Although GUVs were prepared by simple and convenient hydration method in this study, in the future, we would like to prepare more homogeneous GUVs by other methods (droplet transfer, electroformation, microfluidic device methods) to clarify the correlation between GUV structure and membrane fusion properties of the enveloped virus capsids. In addition, since anionic lipid composition may affect membrane fusion, we plan to evaluate in detail the correlation between anionic lipid ratio and membrane fusion activity in the future.

HepG2 cells internalized the cationic enveloped artificial viral capsids through membrane fusion and micropinocytosis. These results indicate that membrane fusion could be induced by interactions between the enveloped artificial viral capsid and cell membrane, without external stimuli. This study could provide insights into the infection mechanism of natural enveloped viruses into the host cell. We aim to construct an artificial infection model for a GUV or host cell of enveloped artificial viral replica equipped with membrane proteins, such as influenza virus-derived hemagglutinin and SARS-CoV-2-derived spike protein. Such artificial virus models can be used as a cell-specific carrier in drug delivery systems.

## Methods

### General

Reversed-phase HPLC was performed at ambient temperature using a Shimadzu LC-6AD Liquid Chromatography system equipped with a UV/vis detector (220 and 551 nm, Shimadzu SPD-10AVvp) and Inertsil WP300 C18 (GL Science) column (250 × 4.6 mm and 250 × 20 mm). MALDI-TOF MS spectra were obtained using the ultrafleXtreme instrument (Bruker Daltonics) in linear/positive mode with α-cyano-4-hydroxy cinnamic acid (α-CHCA) as matrix. Deionized water of high resistivity (> 18 MΩ cm) was purified using Millipore Purification System (Milli-Q water) and used as solvent for the peptides. All reagents were obtained from commercial sources and used without further purification.

### Synthesis of β-annulus-EE peptide

H-Ile-Asn(Trt)-His(Trt)-Val-Gly-Gly-Thr(tBu)-Gly-Gly-Ala-Ile-Met-Ala-Pro-Val-Ala-Val-Thr(tBu)-Arg(Pbf)-Gln(Trt)-Leu-Val-Gly-Ser(tBu)-Glu(OtBu)-Glu(OtBu)-Alko-PEG resin was synthesized on Fmoc-Glu(OtBu)-Alko-PEG resin (494 mg, 0.123 mmol/g; Watanabe Chemical Ind. Ltd.) using Fmoc-based coupling reactions (4 equiv. of Fmoc amino acid). *N*-methylpyrrolidone (NMP) solution containing (1-cyano-2-ethoxy-2-oxoethylidenaminooxy)dimethylamino-morpholino-carbenium hexafluorophosphate (COMU, 4 equiv. of Fmoc amino acid) and diisopropylethylamine (4 equiv. of Fmoc amino acid) was used as coupling reagent. Fmoc deprotection was achieved using 20% piperidine in *N,N*-dimethylformamide (DMF). The progression of coupling and Fmoc deprotection were confirmed by TNBS and chloranil test kit (Tokyo Chemical Industry Co., Ltd.). Peptidyl-resins were washed with NMP and dried under vacuum. Peptides were deprotected and cleaved from the resin with a cocktail of trifluoroacetic acid (TFA):1,2-ethanedithiol:triisopropylsilane:water = 3.76:0.1:0.04:0.1 (mL) at 25 °C for 4 h. Reaction mixtures were filtered to remove resins, and filtrates were concentrated in vacuo. The peptide was precipitated by adding methyl *tert*-butyl ether to the residue and the supernatant was decanted. The sample was washed three times with methyl *tert*-butyl ether, and the precipitated peptide was dried in vacuo. The crude product was purified by reverse-phase HPLC, eluted with a linear gradient of CH_3_CN/water containing 0.1% TFA (5/95–100/0 in 100 min). The fraction containing the desired peptide was lyophilized to prepare 33.5 mg of a flocculent solid (35% yield). MALDI-TOF MS (matrix: α-CHCA, *m/z* = 2564 [M + H]^+^).

### Synthesis of TMR-β-annulus-EE peptide

β-Annulus-EE peptide, bearing Cys at the N-terminal (CINHVGGTGGAIMAPVAVTRQLVGSEE), was synthesized on Fmoc-Glu(OtBu)-Alko-PEG resin (400 mg, 0.1 mmol/g; Watanabe Chemical Ind. Ltd.) using a similar procedure. The crude product was purified by reverse-phase HPLC, eluting with a linear gradient of CH_3_CN:water containing 0.1% TFA (5/95–100/0 over 100 min). The fraction containing the desired peptide was lyophilized to produce 7.4 mg of flocculent solid (29% yield); MALDI-TOF MS (matrix: α-CHCA, *m/z* = 2667 [M]^+^). Cys-β-annulus-EE peptide powder was dissolved in 20 mM phosphate buffer (926 μL, pH 7.0) in an Eppendorf tube and incubated with 38 mM tris(2-carboxyethyl)phosphine hydrochloride (TCEP-HCl, Wako Co., Ltd.) in Milli-Q water (26 μL) and 10.4 mM tetramethylrhodamine-5-maleimide (TMR-maleimide, Funakoshi Co., Ltd.) in 48 μL DMSO in the dark at 25 °C for 7 h (final concentration: 250 μM Cys-β-annulus-EE peptide, 1 mM TCEP-HCl, and 500 μM TMR-maleimide). After dialysis (Spectra/por7, cutoff Mw 1000, Spectrum Laboratories, Inc.) against deionized water for 24 h was complete, the sample was purified by reverse-phase HPLC, eluting with a linear gradient of CH_3_CN/water containing 0.1% TFA (5/95–100/0 over 100 min). The fraction containing the peptide was lyophilized and dissolved in water (100 μL) to prepare an aqueous solution of 129 μM TMR-β-annulus-EE (5.2% yield); MALDI-TOF MS (matrix: α-CHCA, *m/z* = 3149 [M + 2]^+^).

### Preparation of TMR-labeled artificial viral capsid

Briefly, 10 mM phosphate buffer (pH 7.0; 99 μL) was mixed with a stock solution of 10 mM β-annulus-EE peptide (0.9 μL) and 10 mM TMR-β-annulus-EE peptide (0.1 μL) in DMSO in a Eppendorf tube and vortexed for 30 s to prepare TMR-labeled viral capsid (final concentration; [β-annulus-EE] = 90 μM, [TMR-β-annulus-EE] = 10 μM) in 10 mM phosphate buffer containing 1% DMSO (pH 7.0).

### Preparation of TMR/NBD-labeled enveloped viral capsid

Stock solutions of DOTAP in chloroform (10 mM, 3 μL; Avanti Polar Lipids), DOPC in chloroform (10 mM, 29.85 μL; Tokyo Chemical Industry Co., Ltd.), NBD-PE in chloroform (1 mM, 1.5 μL; Avanti Polar Lipids), and chloroform (65.65 μL) were placed in a glass tube and dried in vacuo for 6 h. The resulting lipid film was hydrated with the TMR-labeled artificial viral capsid in 10 mM phosphate buffer (pH 7.0) containing 1% DMSO (90 μM β-annulus-EE, 10 μM of TMR-β-annulus-EE, 100 μL) at 50 °C for 1 h. Excess lipid was removed by ultracentrifugation (25,000 rpm, 2 min, Optima MAX-TL ultracentrifuge, Beckman Coulter) to obtain a solution of TMR/NBD-labeled enveloped viral capsid. TMR-labeled enveloped viral capsid (without NBD labeling) was prepared similarly.

### DLS and ζ-potential

DLS values of samples were measured at 25 °C using Zetasizer Nano ZS (MALVERN) with an incident He–Ne laser (633 nm) and ZEN2112-Low volume glass cuvette cell. During measurements, count rates (sample scattering intensities) were provided. Correlation time for scattered light intensity *G*(*t*) was measured several times, and the averaged results were fitted to Eq. (1), where *B* is the baseline, *A* is the amplitude, *q* is the scattering vector, *t* is the delay time, and *D* is the diffusion coefficient:

1 $$G (\tau ) = B + A \cdot {\mathrm{exp}} (-2{q}^{2}D\tau )$$

The hydrodynamic radius (*R*_*H*_) of the scattering peptides was calculated using Stokes–Einstein equation (Eq. (2)), where *η* is solvent viscosity, *k*_*B*_ is Boltzmann’s constant, and *T* is absolute temperature.

2 $${R}_{H} = \frac{{k}_{B}T}{6\pi \eta D}$$

Zeta potentials of each sample were measured at 25 °C using a Zetasizer Nano ZS (MALVERN) with a DTS1070 clear disposable zeta cell.

### TEM

Cu-grids (thin carbon film TEM grids, ALLIANCE Biosystems) were hydrophilized by plasma treatment using JEOL HD Treatment apparatus for 40 s (60 Hz, 500 VA) at 25 °C. Aliquots (5 μL) of aqueous sample solutions were applied to the hydrophilized Cu-grids for 1 min. Subsequently, the TEM grids were instilled in the staining solution (5 μL) EM stainer (Nisshin-EM) for 10 min. The sample-loaded Cu-grids were dried in vacuo and observed by TEM (JEOL JEM 1400 Plus) at an accelerating voltage of 80 kV.

### Preparation of GUVs^69^

GUVs (a–d) were prepared with a natural swelling method using D-glucose. (a) GUVs containing 100% DOPC or 100% POPC; chloroform/methanol (2/1, v/v) solution of DOPC or POPC (10 mM, 20 μL) was mixed with methanol solution of d-glucose (1 equiv. of lipids). (b) GUVs containing 40% DOPG/DOPC or 40% DOPG/POPC; chloroform/methanol (2/1, v/v) solution of DOPC or POPC (10 mM, 12 μL) and DOPG (10 mM, 8 μL) were mixed with methanol solution of D-glucose (1 equiv. of lipids). (c) GUVs containing 40% GM3/DOPC or 40% GM3/POPC; chloroform/methanol (2/1, v/v) solution of DOPC or POPC (10 mM, 12 μL) and GM3 (5 mM, 16 μL) were mixed with methanol solution of D-glucose (1 equiv. of lipids). (d) NBD/rhodamine-labeled GUV containing 40% DOPG/DOPC or 100% DOPC; chloroform/methanol (2/1, v/v) solution of 40% DOPG/DOPC (DOPG; 10 mM, 8 μL, DOPC; 10 mM, 12 μL) or DOPC (10 mM, 20 μL), NBD-PE (1 mM, 1.5 μL), and 1,2-dioleoyl-*sn*-glycero-3-phophoethanolamine-*N*-(lissamine rhodamine B sulfonyl) (rhodamine-PE, 1 mM, 3 μL; Avanti Polar Lipids) were mixed with methanol solution of D-glucose (1 equiv. of lipids). These solutions were dried in a glass test tube (i.d. ~ 1 cm) in vacuo for 6 h. The dried film was hydrated at 50 °C with 10 mM phosphate buffer (pH 7.0) (100 μL) for 1 h.

### Interaction of enveloped viral capsids with GUVs

A solution of TMR/NBD-labeled enveloped viral capsids in 10 mM phosphate buffer (pH 7.0) containing 1% DMSO (1 μL) was incubated with the aqueous suspension of GUVs (1 μL) at 25 °C for 1 h. Fluorescence was observed with a CLSM using FluoView FV10i (Olympus). TMR was excited with 553 nm and observed through a 577 nm emission band-pass filter (red). NBD-PE was excited with 473 nm and observed through a 490–540 nm emission band-pass filter (green). Relative fluorescence intensity of TMR in GUVs was calculated by dividing fluorescence intensity inside GUVs by fluorescence intensity outside GUVs using ImageJ software (*N* = 20 images for GUVs).

### Lipid-mixing assay^66^

A solution of enveloped viral capsids (50 μM β-annulus-EE peptide, 150 μM DOTAP, and 1500 μM DOPC) in 10 mM phosphate buffer (pH 7.0) containing 1% DMSO (1 μL) was incubated with the aqueous suspension of TMR/rhodamine-labeled 40% DOPG/DOPC or DOPC GUVs (1 μL) at 25 °C for 1 h. Fluorescence was observed with a CLSM using FluoView FV10i. NBD and rhodamine were excited with 489 nm and observed through 510 nm and 610 nm emission band-pass filter (green; NBD, red; rhodamine).

### Interaction of enveloped viral capsids with HepG2 cells

HepG2 cells (RIKEN BioResource Research Center, Japan) were cultured in Dulbecco’s modified Eagle’s medium (DMEM). All medium contained 10% fetal bovine serum (v/v), 100 μg/mL streptomycin, 100 U/mL penicillin, 1 mM sodium pyruvate, and 1% MEM nonessential amino acids (v/v, Sigma M7145). Cells were maintained at 37 °C in a 5% CO_2_ incubator, and a subculture was performed every 3–4 days. HepG2 cells were seeded onto a single-well glass-bottom dish at 2.0 × 10^4^ cells/ well in a final volume of 100 μL, and incubated for 24 h at 37 °C in 5% CO_2_. The cells were incubated with a solution (80 μL) of TMR/NBD-labeled enveloped viral capsid (45 μM β-annulus-EE peptide, 5 μM TMR-β-annulus-EE peptide, 150 μM DOTAP, 1492.5 μM DOPC and 7.5 μM NBD-PE) in DMEM (−) for 5 min, 1 h, and 3 h at 37 °C in a 5% CO_2_ incubator. The solution was removed, and the cells were incubated with 10 μg/mL Hoechst 33342 (80 μL) for 10 min at 37 °C in 5% CO_2_. The cells were washed with PBS and the medium was added to the cells. Fluorescence was observed by CLSM. NBD-PE was excited with 473 nm and observed through a 490–540 nm emission band-pass filter (green). TMR was excited with 553 nm and observed through a 577 nm emission band-pass filter (red). Hoechst 33342 was excited with 352 nm and observed through a 455 nm emission band-pass filter (cyan).

### Endocytosis inhibition study

HepG2 cells were seeded onto a single-well glass-bottom dish at 2.0 × 10^4^ cells/well in a final volume of 100 μL and incubated for 24 h at 37 °C in 5% CO_2_. The cells were incubated with endocytosis inhibitors 80 μM EIPA (Funakoshi Co., Ltd.), 160 μM genistein (Funakoshi Co., Ltd.), and 20 μM Pitstop 2 (Sigma-Aldrich) in DMEM (−) for 30 min at 37 °C in 5% CO_2_ and washed with PBS. The cells were treated with a solution of TMR-labeled enveloped viral capsid (60 μL) containing 80 μM EIPA, 160 μM genistein, or 20 μM Pitstop 2 in DMEM (−) for 3 h at 37 °C in 5% CO_2_ and washed with PBS. The medium was added to the cells and fluorescence was observed by CLSM. TMR was excited with 553 nm and observed through a 577 nm emission band-pass filter (red). Relative fluorescence intensity of TMR in cells was measured by subtracting background intensity using ImageJ software (*N* = 50).

### Supplementary Information

Supplementary Figures.

## Data Availability

All data generated and analyzed during this study are included within this published article or its supplementary information.
