# Supplementary material for: A supramolecular system mimicking the infection process of an enveloped virus through membrane fusion
Source: Sci Rep. 2023 Nov 15;13:19934. doi: 10.1038/s41598-023-47347-7 (PMC10651892; doi:10.1038/s41598-023-47347-7)
Supplement: Supplementary file 1 — Supplementary Figures. [file 41598_2023_47347_MOESM1_ESM.docx]

Supplementary Information

**A supramolecular system mimicking the infection process of an enveloped virus through membrane fusion**

**Hiroto Furukawa^1^, Yuuna Kimura^1^, Hiroshi Inaba^1,2^, Kazunori Matsuura^1,2^***

^1^Department of chemistry and biotechnology, Graduate School of Engineering, Tottori University, 4-101 Koyama-Minami, Tottori 680-8552, Japan

^2^Center for Research on Green Sustainable Chemistry, Tottori University, 4-101 Koyama-Minami, Tottori 680-8552, Japan

***** ma2ra-k@tottori-u.ac.jp


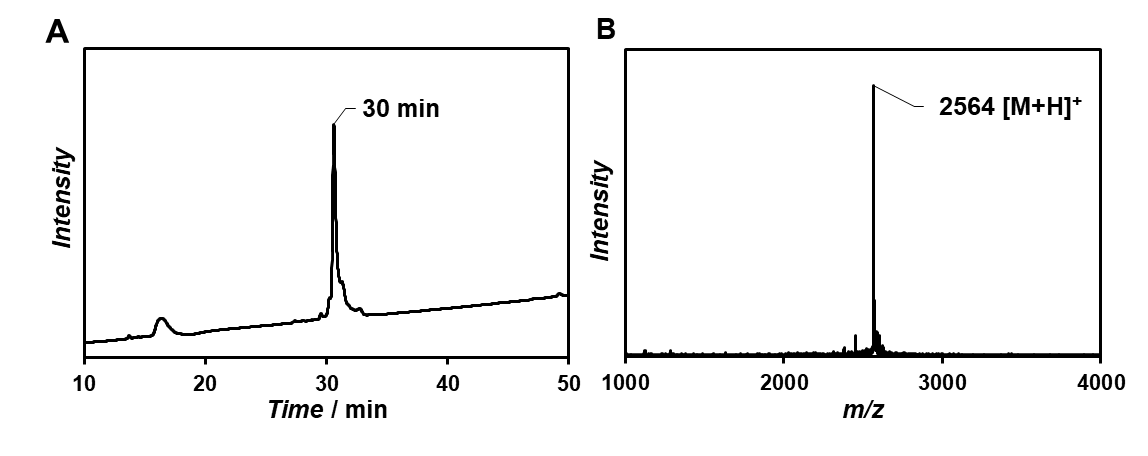


**Figure S1.** (A) HPLC chart and (B) MALDI-TOF-MS of *β*-annulus-EE (matrix: α-CHCA)


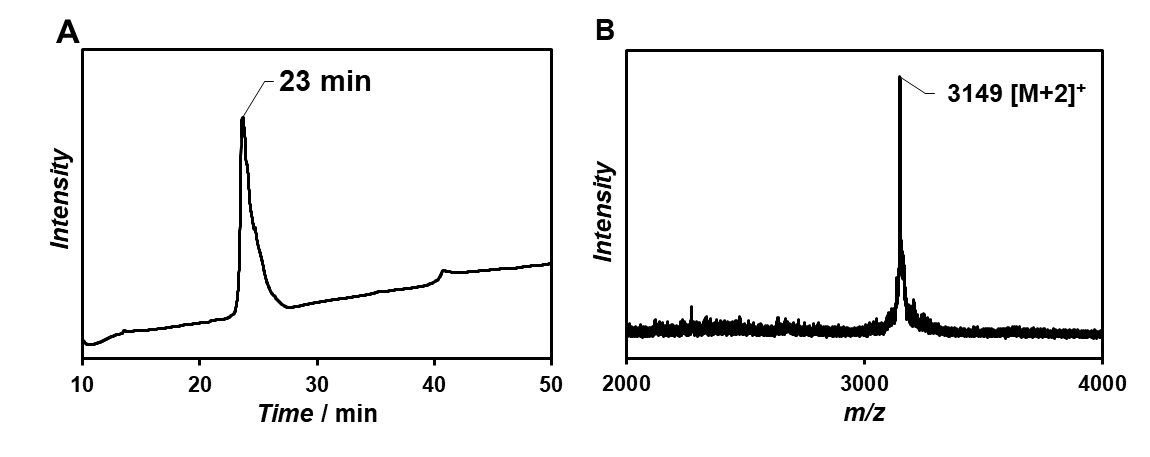


**Figure S2.** (A) HPLC chart and (B) MALDI-TOF-MS of TMR-*β*-annulus-EE (matrix: α-CHCA)


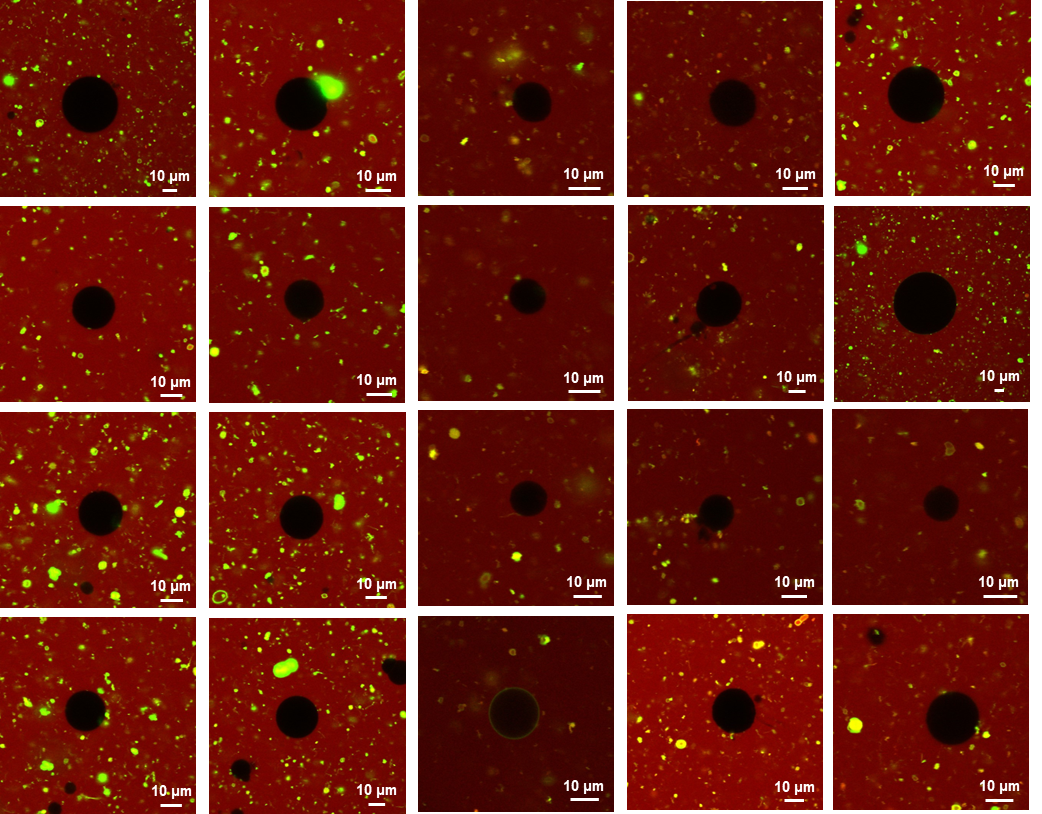


**Figure S3.** CLSM images of DOPC GUV (1 mM DOPC) interacting with TMR/NBD-labeled enveloped viral capsid (45 μM *β*-annulus-EE peptide, 5 μM TMR-*β*-annulus-EE peptide, 150 μM DOTAP, 1492.5 μM DOPC and 7.5 μM NBD-PE).


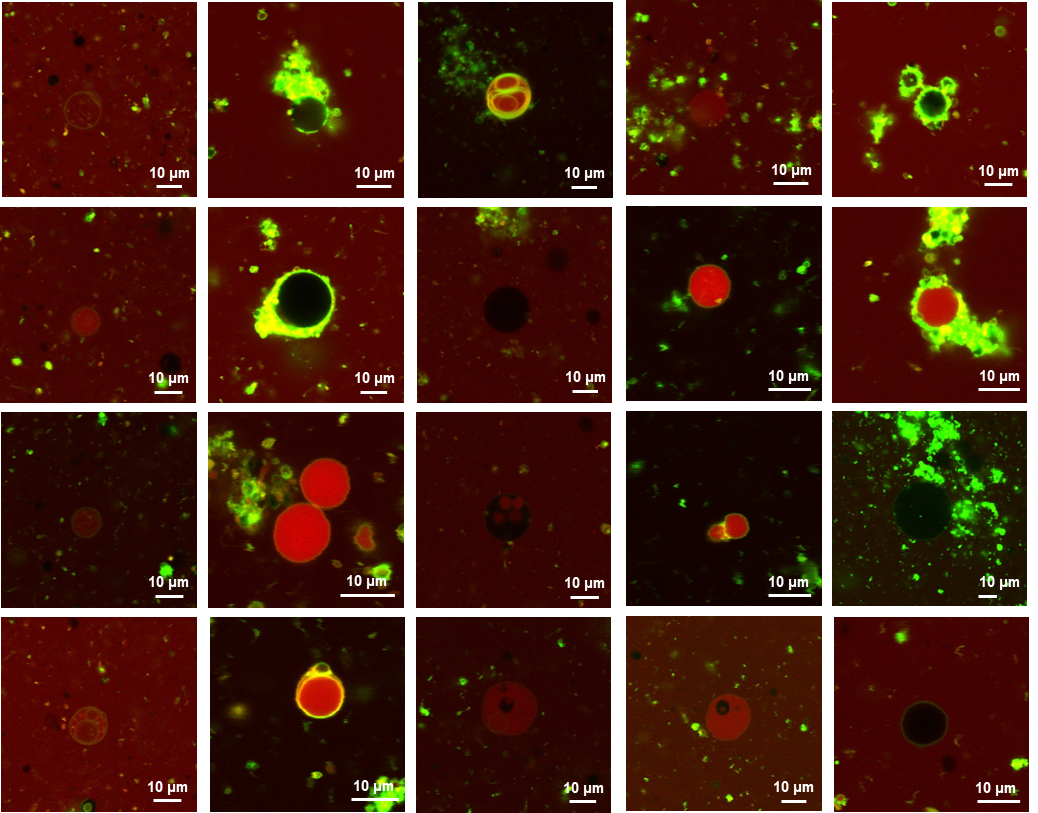


**Figure S4.** CLSM images of 40% DOPG/DOPC GUV (0.4 mM DOPG, 0.6 mM DOPC) interacting with TMR/NBD-labeled enveloped viral capsid (45 μM *β*-annulus-EE peptide, 5 μM TMR-*β*-annulus-EE peptide, 150 μM DOTAP, 1492.5 μM DOPC and 7.5 μM NBD-PE).


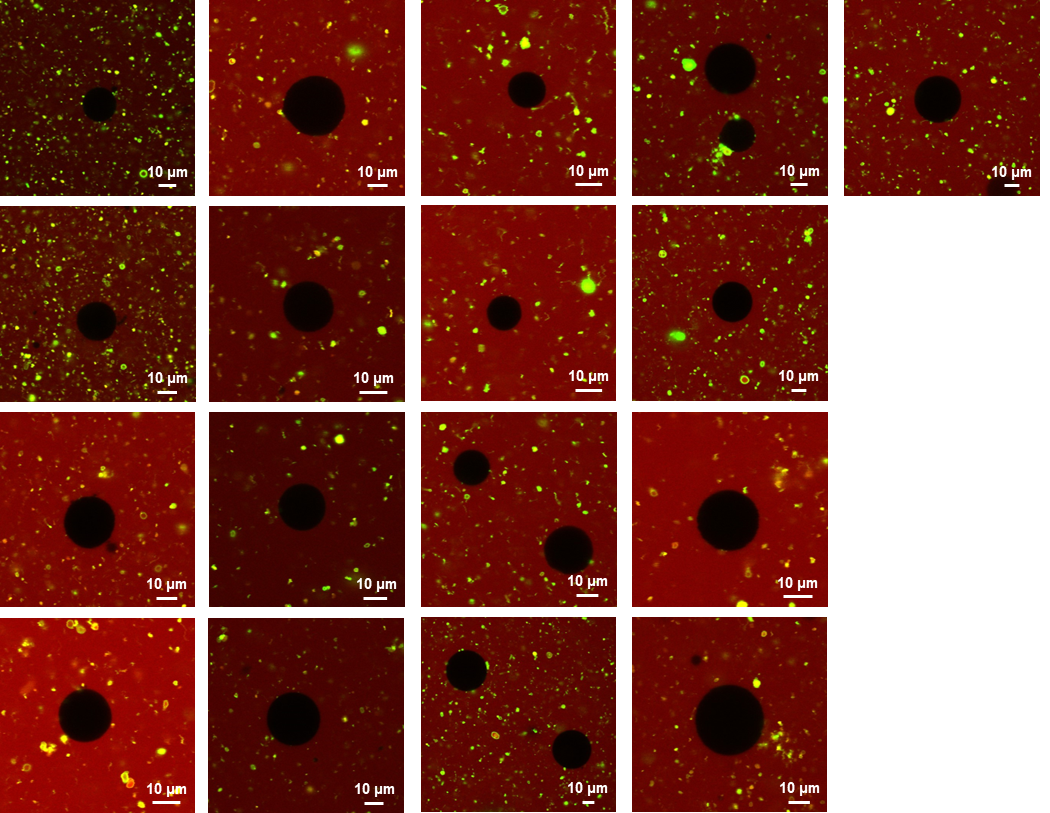


**Figure S5.** CLSM images of POPC GUV (1 mM POPC) interacting with TMR/NBD-labeled enveloped viral capsid (45 μM *β*-annulus-EE peptide, 5 μM TMR-*β*-annulus-EE peptide, 150 μM DOTAP, 1492.5 μM DOPC and 7.5 μM NBD-PE).


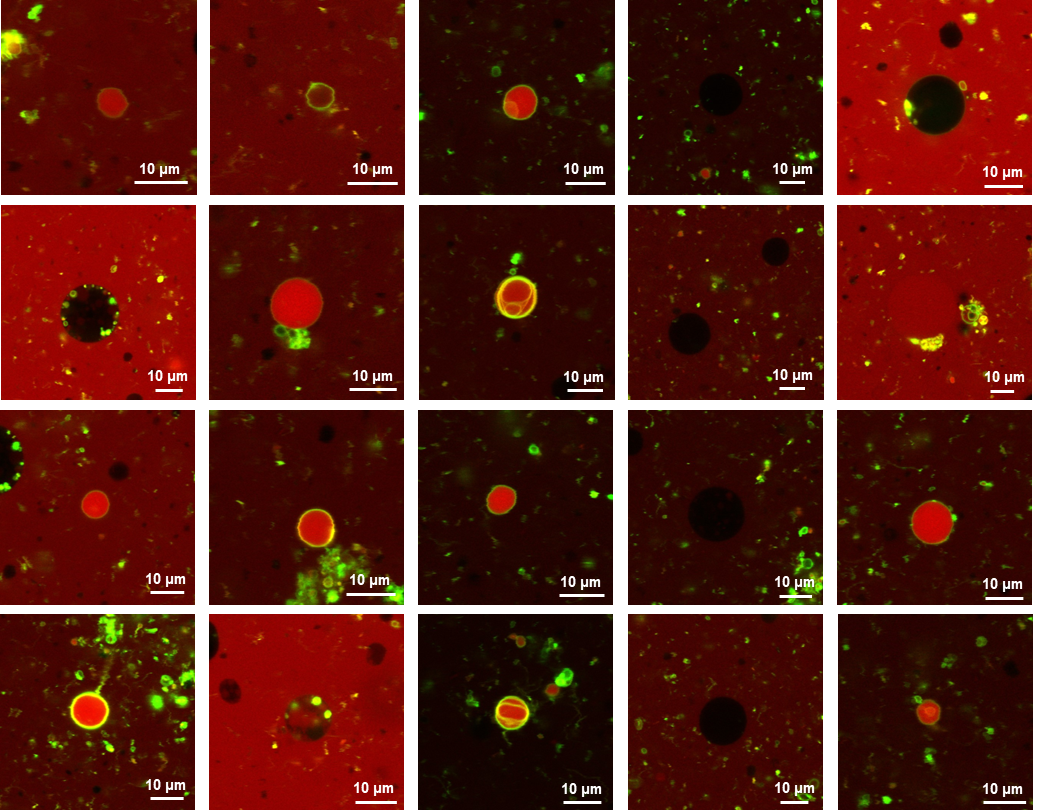


**Figure S6.** CLSM images of 40% DOPG/POPC GUV (0.4 mM DOPG, 0.6 mM POPC) interacting with TMR/NBD-labeled enveloped viral capsid (45 μM *β*-annulus-EE peptide, 5 μM TMR-*β*-annulus-EE peptide, 150 μM DOTAP, 1492.5 μM DOPC and 7.5 μM NBD-PE).


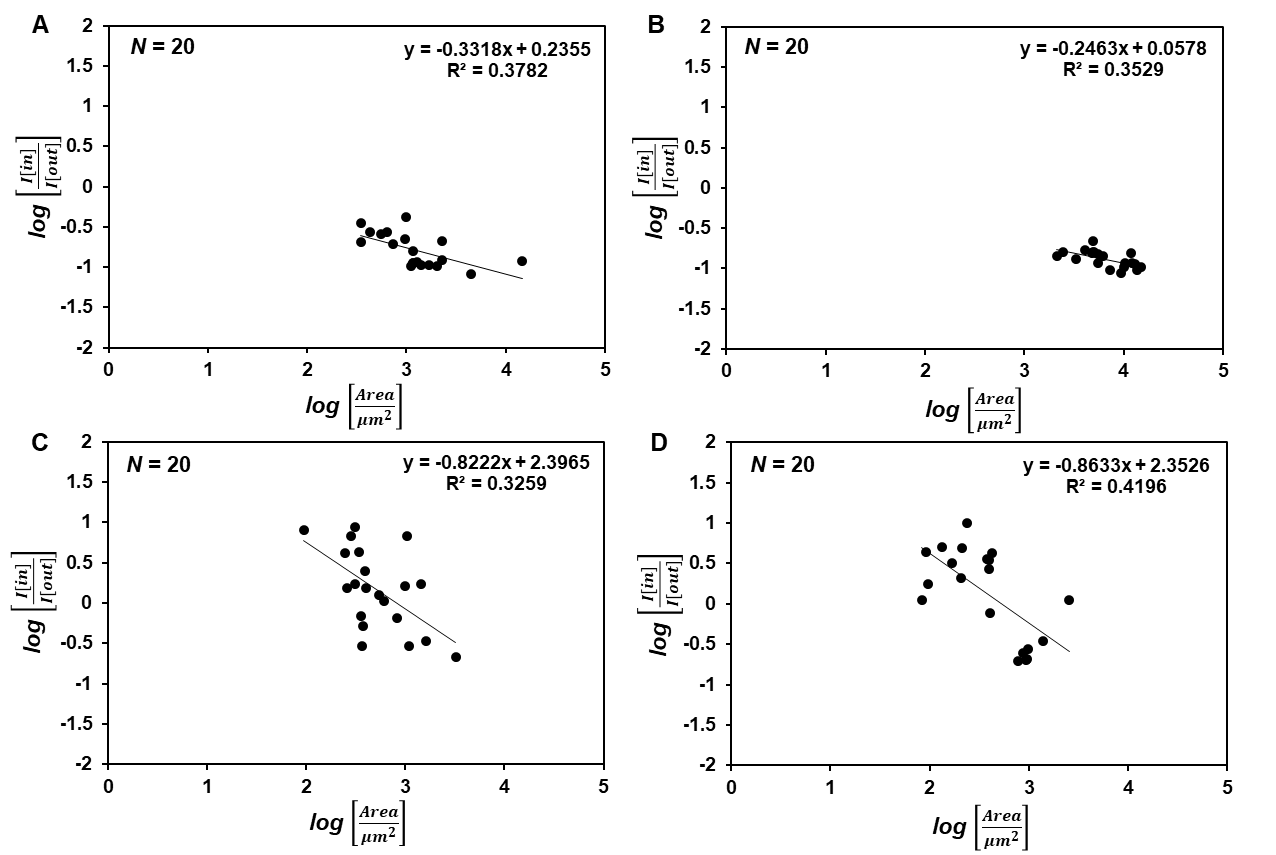


**Figure S7.** GUV surface area dependence of the fluorescence intensity ratio of TMR inside/outside of (A) DOPC GUV (1 mM DOPC), (B) POPC GUV (1 mM POPC), (C) 40% DOPG/DOPC GUV (0.4 mM DOPG, 0.6 mM DOPC), 40% DOPG/POPC GUV (0.4 mM DOPG, 0.6 mM POPC) approximated by a power law equation (*N* = 20).


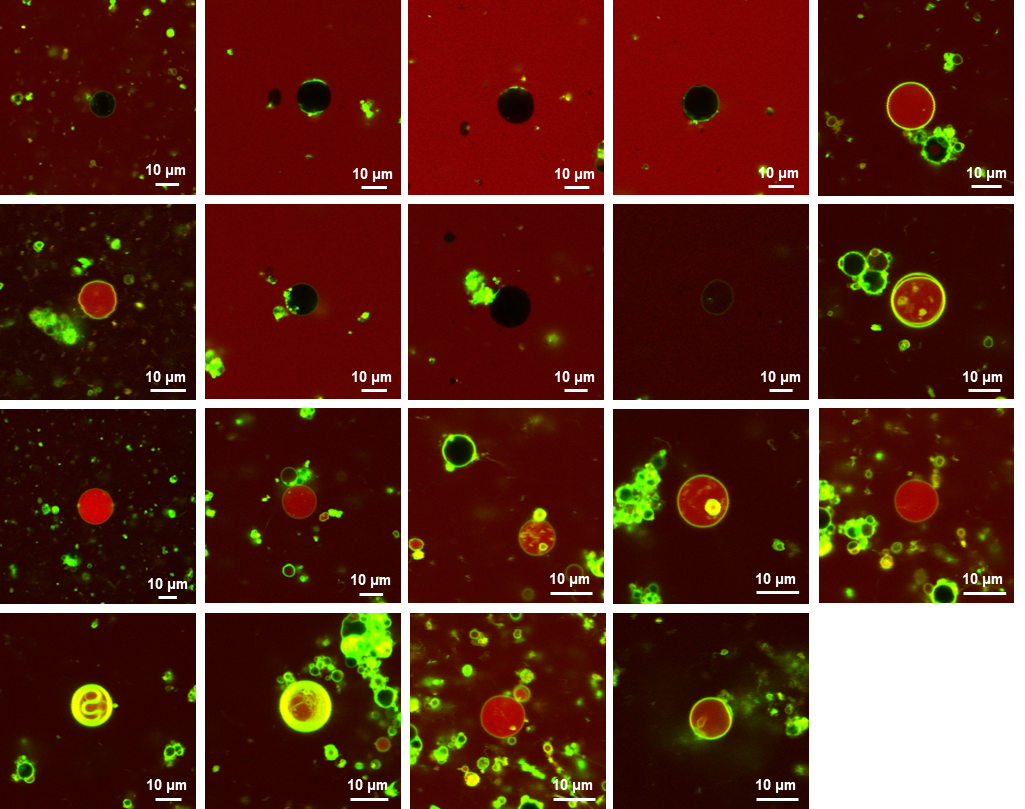


**Figure S8.** CLSM images of 40% GM3/DOPC GUV (0.4 mM GM3, 0.6 mM DOPC) interacting with TMR/NBD-labeled enveloped viral capsid (45 μM *β*-annulus-EE peptide, 5 μM TMR-*β*-annulus-EE peptide, 150 μM DOTAP, 1492.5 μM DOPC and 7.5 μM NBD-PE).


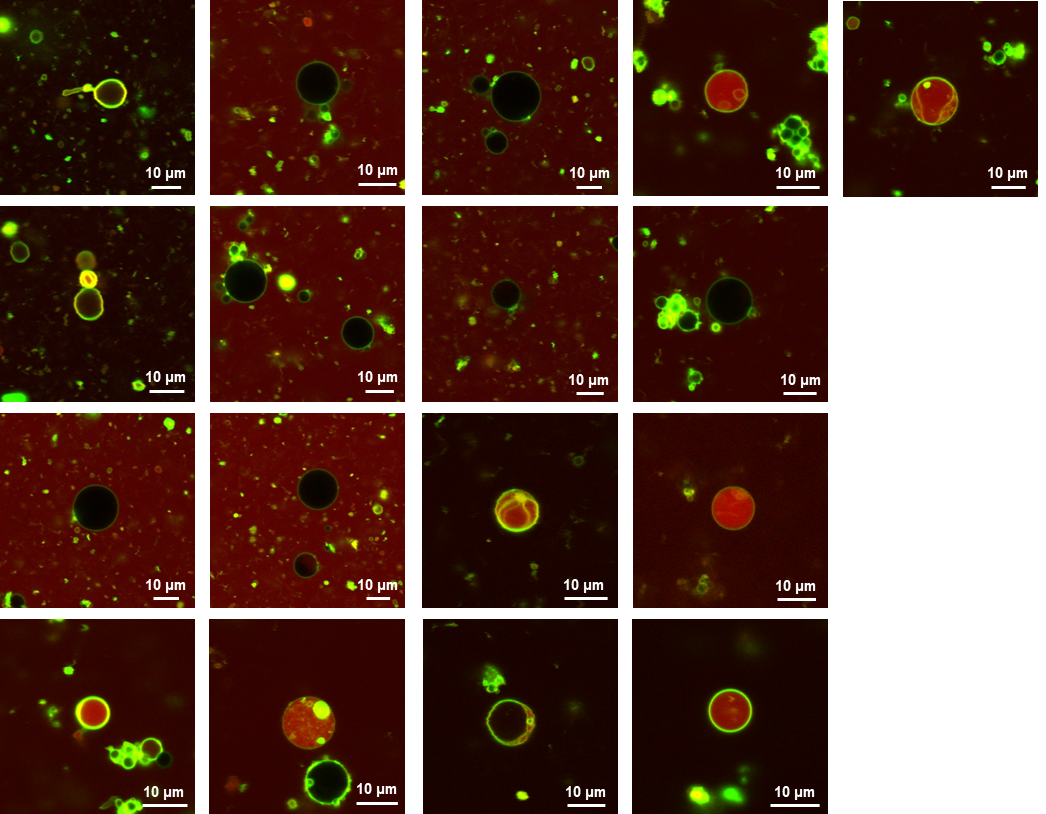


**Figure S9.** CLSM images of 40% GM3/POPC GUV (0.4 mM GM3, 0.6 mM POPC) interacting with TMR/NBD-labeled enveloped viral capsid (45 μM *β*-annulus-EE peptide, 5 μM TMR-*β*-annulus-EE peptide, 150 μM DOTAP, 1492.5 μM DOPC and 7.5 μM NBD-PE).


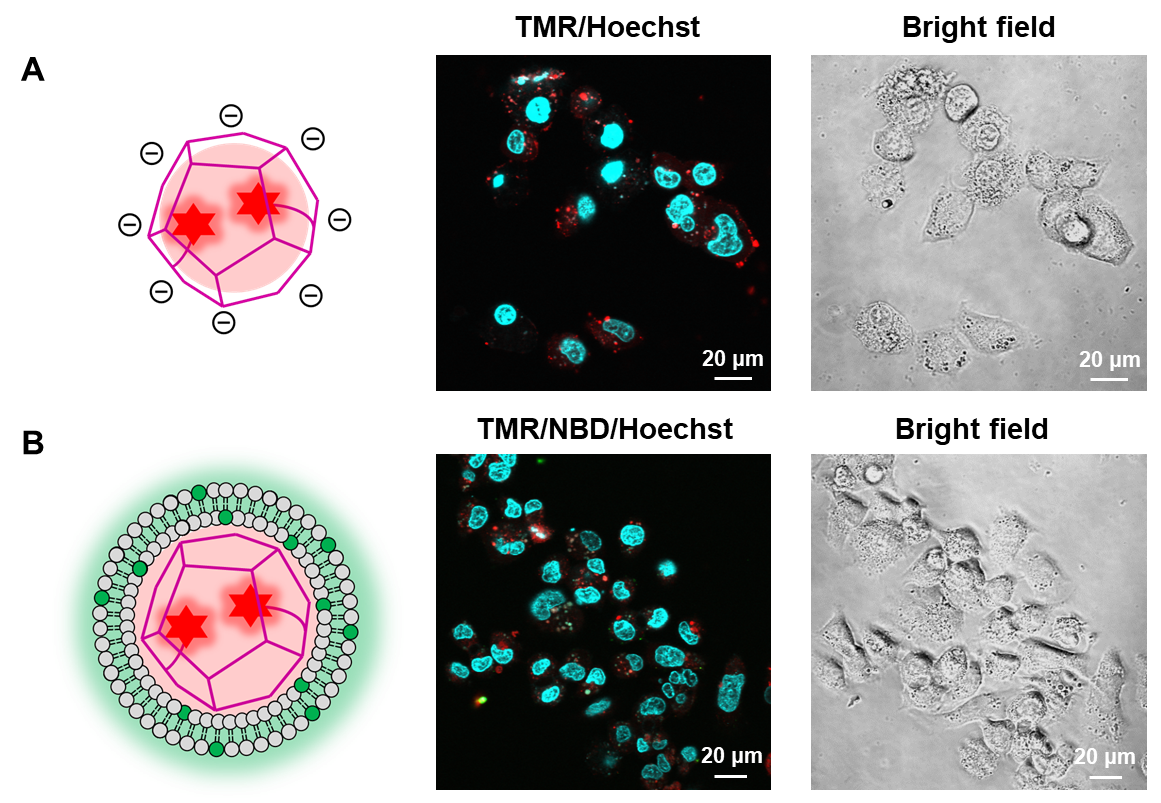


**Figure S10.** CLSM images of HepG2 cells incubated with (A) TMR-labeled viral capsid (45 μM *β*-annulus-EE peptide, 5 μM TMR-*β*-annulus-EE peptide) and (B) TMR/NBD-labeled neutral enveloped viral capsid (45 μM *β*-annulus-EE peptide, 5 μM TMR-*β*-annulus-EE peptide, 1492.5 μM DOPC, 7.5 μM NBD-PE) for 3 h. Nuclei were stained with Hoechst 33342.


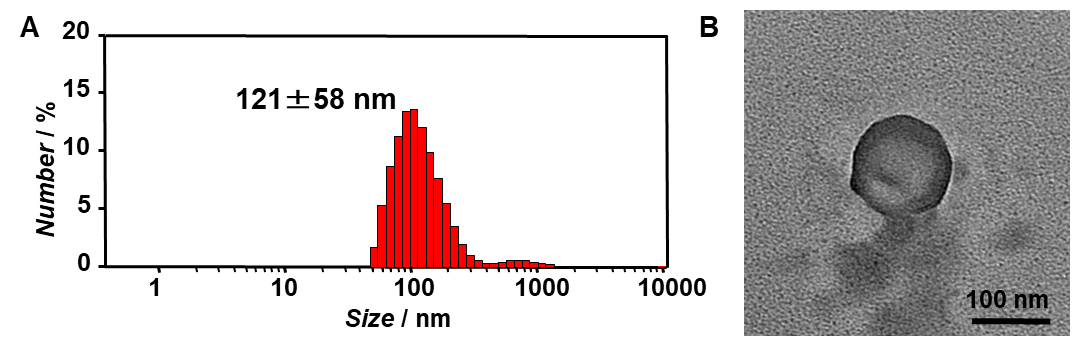


**Figure S11.** (A) Size distribution obtained from DLS and (B) TEM image of TMR-labeled enveloped viral capsid (90 μM *β*-annulus-EE peptide, 10 μM TMR-*β*-annulus-EE peptide, 300 μM DOTAP, 3000 μM DOPC) in phosphate buffer (pH 7.0) at 25 ºC. TEM sample was stained with EM stainer.
